# Stoichiometry of Bulk Nb_1−β_Sn_β_ Superconductors Synthesised by Arc Melting

**DOI:** 10.3390/ma18133050

**Published:** 2025-06-27

**Authors:** Mahboobeh Shahbazi, Henrietta E. Cathey, Ali Dehghan Manshadi, Jose Alarco, Ian D. R. Mackinnon

**Affiliations:** 1 Centre for Materials Science and School of Chemistry and Physics, Queensland University of Technology (QUT), Brisbane, QLD 4001, Australia; mahboobeh.shahbazi@qut.edu.au (M.S.); jose.alarco@qut.edu.au (J.A.); 2 Centre for Clean Energy Technologies and Practices, Queensland University of Technology (QUT), Brisbane, QLD 4001, Australia; 3 School of Earth and Atmospheric Sciences, Queensland University of Technology (QUT), Brisbane, QLD 4001, Australia; henrietta.cathey@qut.edu.au; 4 School of Mechanical and Mining Engineering, The University of Queensland, Brisbane, QLD 4072, Australia; aliuow@gmail.com

**Keywords:** superconductor, bulk Nb_3_Sn, arc melting, stoichiometry, critical current density, microstructures

## Abstract

We present an alternative process for production of binary Nb_1−β_Sn_β_ superconducting phases using pre- and post-treatment of arc-melted Nb + Sn ingots. This process combines sequential sintering, arc melting, and annealing procedures that provide dense, bulk samples of Nb_1−β_Sn_β_ with varying stoichiometry between 0.18 < β < 0.25 depending on annealing time and temperature. We show, through magnetization measurements of these Nb_1−β_Sn_β_ bulks, that annealing of arc-melted samples at 900 °C for 3 h significantly enhances *J*_c_ values compared with arc-melted Nb_1−β_Sn_β_ samples without annealing. Microstructural analyses show that optimum grain size and orientation are achieved by sintering and annealing at lower temperatures (i.e., 720 °C and 900 °C, respectively) with short annealing times (i.e., <10 h). Processing at higher temperatures and for longer times enhances grain growth and results in fewer pinning centres. The optimum process creates effective pinning centres that deliver a *J*_c_ = 6.16 × 10^4^ A/cm^2^ at 10 K (and ~0.2 T), compared with *J*_c_ = 3.4 × 10^4^ A/cm^2^ for Nb_1−β_Sn_β_ subjected to a longer annealing time at a higher temperature and *J*_c_ = 775 A/cm^2^ for an arc-melted sample without post-annealing. We suggest that further work addressing post-treatment annealing times between 3 h < t_post_ < 60 h at temperatures between 900 °C and 1000 °C will provide the opportunity to control stoichiometric and microstructural imperfections in bulk Nb_1−β_Sn_β_ materials.

## 1. Introduction

Nb_3_Sn was discovered more than half a century ago [1] and continues to hold a crucial role in applications involving high-field magnets. These applications include nuclear magnetic resonance spectroscopy [2], future circular colliders [3], and the International Thermonuclear Experimental Reactor (ITER) [4]. Nb_3_Sn is an intermetallic compound of niobium and tin with an A15-type structure that enables superconductivity without anisotropy in wire applications [5]. Compared with common low-temperature superconducting materials, Nb_3_Sn has a higher *J*_c_ and higher *T*_c_, but it is very brittle compared with ductile NbTi material [6].

The primary use of Nb_3_Sn is as superconducting wires for high-magnetic-field applications [7]. Effective application has built upon fundamental studies [5,8] of the binary Nb–Sn system and recognition that wire manufacture requires utilization of a ternary system such as Nb–Cu–Sn or Nb–Ti–Sn [5,7,9,10,11]. For example, the bronze route to synthesis using Cu enables production of Nb_1−β_Sn_β_ compositions (including Nb_3_Sn) by moderating the chemical potential of component elements, particularly that of Sn [12]. An excellent summary of the reaction behaviors of Nb_3_Sn in wire processing via the bronze route and by other techniques is provided by Banno [12].

The synthesis of binary Nb_3_Sn as bulk material is a challenge due to the high diffusivity of Sn and other phases such as NbSn_2_, with consequent departure from Fick’s law [12]. These challenges led Devantay et al. [8], Goldacker et al. [13], and others to explore novel syntheses to produce homogeneous bulk Nb_3_Sn, for example, by using levitation melting at high Argon pressure [8] or by utilizing a hot isostatic press (HIP) at 1100 °C [13]. To further minimize inhomogeneity, Zhou et al. [14] also employed an HIP process capable of reaching temperatures up to 2200 °C in combination with ball milling and pre-annealing of starting materials.

Arc melting is an alternative synthesis technique to prepare metals, alloys, or intermetallics in bulk amounts with minimal contamination or introduction of impurities [15]. The process offers rapid in situ melting to very high temperatures, rapid solidification with minimal element losses, and uniform distribution of constituents [15]. The technique is readily available for laboratory use and is a well-established, scalable industrial process for many alloys and intermetallics [15]. For example, arc melting is used in steel manufacture [16,17], and for production of biomedical titanium alloys [18] and composite materials [19,20].

In this work, we evaluate pre-treatment preparation, arc melting of pre-treated samples, and annealing times before and after arc melting to explore their suitability for effective binary production of bulk Nb_3_Sn material. To determine the stoichiometry of specific phases in these bulk samples, we used high spatial and analytical precision electron microprobe analyses in addition to spatially resolved crystallographic parameters that enabled sub-micrometer delineation of the microstructure and phase relations. This work focuses on approaches amenable to bulk formation, while aiming for high homogeneity of the microstructure in order to expand the scope of potential applications for shaped Nb_3_Sn.

## 2. Materials and Methods

A molar ratio of niobium powder (45 μm, 99.8% purity) and tin powder (<325 mesh size, 99.8% purity) supplied by Sigma-Aldrich (Ryde, NSW, Australia) was weighed, ground in an agate mortar in an Argon-filled glove box, and pressed into a pellet (diameter ~1 cm × depth 5 mm) under an applied pressure of 10 tonne for 2 min. Since the difference in the melting temperature of Sn and Nb is large, arc melting may cause deviations from the intended stoichiometry due to vaporization or loss of Sn. Therefore, pre-annealing was performed on all samples to reduce the potential for Sn evaporation.

The pellet(s) for pre-annealing were placed inside a boron nitride sleeve within a 100 mL Parr reactor using a controlled atmosphere glove box containing Argon (99.99% purity). The reactor was then sealed tightly and removed from the glove box for operation. Two sets of samples were prepared using a mixture of 3:1 stoichiometric Nb + Sn, for heat treatment under an Ar atmosphere at a heating rate of 2 °C/minute to (i) 720 °C held for 105 h and (ii) to 1000 °C held for 60 h. The reactor temperature was thermocouple-controlled to ±2 °C.

These sintered pellets were then melted in an arc furnace on a water-cooled copper hearth under argon. The use of a water-cooled hearth with the arc melting technique helped reduce contamination from the crucible that contained the starting mixture and allowed easy removal of oxygen from the surrounding gas in the arc furnace. Prior to the arc-melting experiments, the chamber was vented and filled with argon three times and then filled with argon in order to eliminate oxygen in the chamber. The argon atmosphere was further purified by melting zirconium foam before melting the Nb-Sn pellet. The sample mixture was melted three times to ensure complete mixing. The arc-melted samples (diameter ~1 cm × 5 mm depth) were then annealed at 900 °C for 3 h; the second set of samples was annealed at 1000 °C for 60 h. All samples were subjected to the same annealing process (except for the time and temperature conditions) as described above for the pre-annealing process. Table 1 lists the sample IDs and relevant sintering and/or annealing time/temperature of the samples. After synthesis, ingots were split into representative pieces to enable phase, microstructural, and physical properties analyses.

All samples were subjected to x-ray powder diffraction (XRD) analysis using a Bruker D8 Advance X-ray diffractometer (Billerica, MA, USA) with Co Kα1 radiation in Bragg Brentano geometry. XRD measurements were performed with a step size of 0.02° 2θ and a counting time of 10 s per step. Diffraction patterns were refined and indexed using the software program Topas Version 6. Rietveld refinements using Topas determine quantitative estimates of phase abundance in each product. In general, phase abundances determined by this technique are within <5% relative error.

The microstructures of sintered, arc-melted, and annealed specimens were examined using scanning electron microscopy (SEM) equipped with microanalysis. Microstructural features were examined on polished samples using a field emission JEOL 7001SEM (JEOL Australasia Pty Ltd., French’s Forest, Australia), equipped with a secondary X-ray detector (Oxford Instruments SDD XMax 50 mm^2^, Abington, UK) and an electron backscatter diffraction (EBSD) pattern analyzer with Channel 5 analysis software and automated feature detection. Finely polished samples mounted in conductive resin were selected for EBSD mapping using an accelerating voltage of 25 kV and a step size of 0.2 µm.

Quantitative elemental analyses were performed using a JEOL JXA 8530F field emission electron probe microanalyzer (FE-EPMA, JEOL, Tokyo, Japan) equipped with five wavelength-dispersive spectrometers (WDS) and Probe for EPMA Version 13.9.1 software (Eugene, OR, USA). Operating conditions were 10 kV accelerating voltage with a beam current of 40 nA and a fully focused beam. Wavelength-dispersive analyzing crystals were PETL for Nb Lα and PETH for Sn Lα, and calibration standards included pure Nb and Sn metals. On- and off-peak counting times were 30 s, and a linear off-peak correction method was employed for background positions selected on either side of the analytical peaks.

Average detection limits (single spot, 3-sigma) were 360 ppm for Nb and 390 ppm for Sn, with relative analytical sensitivity (at the 99% confidence interval) of 0.3%. Estimated spatial resolution for each analysis under the EPMA operating conditions was ≤0.8 μm, based on Monte Carlo simulations of characteristic X-ray generation volumes in Nb_3_Sn (using density = 8.7) with CASINO Version 2.42 software [21]. For these analyses, the samples were combined with conductive resin and positioned in a 30 mm diameter mould within a hot mounting press. The mounted samples were then polished using a sequence of diamond pads and cloths to achieve a mirror-like finish, ideal for electron microprobe analysis.

Zero-field-cooled (ZFC) and field-cooled (FC) magnetization (*M*) curves were recorded as a function of temperature at 10 Oe using a Cryogenic Ltd. Mini Cryogen-free System (Cryogenic Ltd., London, UK). The system consisted of the following:

- A pulse tube cryocooler with base temperature below 3 K and cooling power 0.5–1 W at 4.2 K;
- A high-stability 20 bit magnet power supply;
- A 5 Tesla magnet;
- A variable temperature sample space of 25 mm ID, 100 mm long isothermal region;
- A cryocooler for both the magnet and the sample space so that neither liquid nitrogen nor liquid helium was required.

Values for *T*_c_ were determined by finding the intersection of the linearly extrapolated *M*(*T*) with a constant *M* line. Magnetic hysteresis loops were measured at several temperatures below T_c_ for all samples. A magnetic *J*_c_ was calculated for samples using the Bean model: *J*_c_ = 20 Δ*M*/[*a*(1 − *a*/3*b*)] (*a* < *b*), where *a* and *b* are the width and length of the sample (in cm) perpendicular to the applied field, respectively. Δ*M* (in emu/cm^3^) is the width of the magnetic hysteresis loop and represents the difference in magnetization between the increasing and decreasing branches of the applied magnetic field and provides a value for *J_c_* in A/cm^2^. Polichetti et al. [22] provide valuable discussion of the proper and improper use of formulae relevant to the Bean model. The formula used in this work followed that described as case study III in Table 3 of reference [22].

## 3. Results

Table 1 summarizes information regarding selected sample IDs, synthesis techniques, and heat-treatment conditions for the Nb_3_Sn bulks reported in this study. For instance, the sample ID “NS-1000/60” represents a Nb + Sn mixture sintered at 1000 °C for 60 h with no arc-melting process, while the ID “NS-1000/60/arc” represents a sample that has been subject to arc melting after a sintering process at 1000 °C for 60 h.

Two sintering temperatures, at 720 °C and 1000 °C, are evaluated in this work to explore the effects of both lower and higher sintering temperature. Preliminary evaluation showed that a Nb–Sn phase did not form with sintering at 720 °C. Nevertheless, NS-720/105/arc/ann/900/3 was prepared from an arc-melted sample where Nb + Sn mixtures were sintered at 720 °C for 105 h before arc melting, followed by annealing at 900 °C for 3 h. The sample ID NS-1000/60/arc/ann/1000/60 represents a sintered sample at 1000 °C for 60 h followed by arc melting and annealing treatment at 1000 °C for 60 h.

### 3.1. Crystallography and Phase Relations

**Figure 1:**
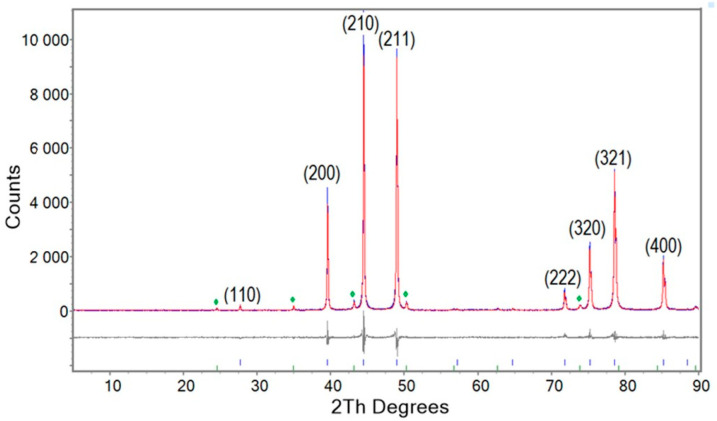
Rietveld refinement of the XRD pattern for NS-1000/60/arc/ann/1000/60 (Run 3). The blue, red, and grey colours represent experimental, refined, and differences in models, respectively. Reflections for Nb_3_Sn are indexed. The green symbols indicate peaks for NbO.

Results from Rietveld refinement to quantitatively determine phase abundances are shown in Figure 1 for the XRD pattern from NS-1000/60/arc/ann/1000/60 (Run 3). The blue, red, and grey colours represent experimental, refined, and differences in models, respectively. As shown in Figure 1, Nb_3_Sn was the predominant phase (94.88%) with denoted indexed reflections, while a minor proportion of secondary phase such as niobium oxide was also observed (green symbols). Similar refinements were performed for all other samples, including those with a high proportion of Nb_3_Sn phase.

Table 2 shows the relative proportions of Nb_3_Sn and other products obtained from selected syntheses with a high proportion of Nb_3_Sn phase according to indexed XRD patterns. Refined XRD data including change in lattice parameters, weighted R factor (R_wp_), and goodness of fit (gof) for each refinement are also shown in Table 2. Run 4 is not included in Table 2 because diffraction from a Nb_3_Sn phase was not observed. The sample from Run 5 was damaged during preparation for characterization and is also not included in Table 2.

In all cases, Nb_3_Sn was the major phase (in proportions above 85%) with minor amounts of other compounds including Nb and NbO. An increase in reaction time for NS-1000/60/arc/ann/1000/60 (Run 3) improves the yield of Nb_3_Sn due to complete conversion of excess Nb. The lattice parameter *a* decreased for arc-melted and annealed samples compared with a sample sintered at 1000 °C for 60 h (NS-1000/60; Run 1). Note the relatively high proportions of unreacted Nb for samples NS-1000/60/arc (Run 2) and NS-720/105/arc/ann/900/3 (Run 6).

### 3.2. Microstructures and Grain Compositions

**Figure 2:**
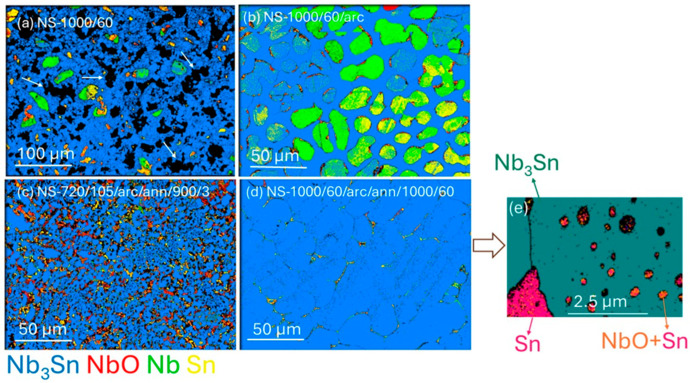
EBSD phase maps of Nb_1−β_Sn_β_ aggregates showing (**a**) NS-1000/60 (Run 1), (**b**) NS-1000/60/arc (Run 2), (**c**) NS-720/105/arc/ann/900/3 (Run 6), (**d**) NS-1000/60/arc/ann/1000/60 (Run 3), (**e**) higher-magnification EBSD phase map of small black areas for NS-1000/60/arc/ann/1000/60. The white arrows in (**a**) denote holes in NS-1000/60. Colour scheme for (**a**–**d**) matches the compound legend below (**c**): Nb_3_Sn—blue; NbO—red; Nb—green; Sn—yellow. (**e**) Nb_3_Sn—green; Sn—red; NbO + Sn—orange plus red.

EBSD phase maps of Nb_3_Sn bulks for sintered, arc-melted, and annealed samples are shown in Figure 2. For all samples, Nb_3_Sn was the major phase and XRD Rietveld analyses confirmed that >85% of all samples were Nb_3_Sn. According to EBSD analysis, minor phases of Nb, Sn, and NbO were present in all samples. Diffraction peaks for Sn were not detected in the XRD patterns. The discrepancy in detecting Sn by diffraction methods may have been due to a localized presence on the surface of samples, low concentration, or an amorphous structure. In the latter case, Sn was detectable via EBSD but not by using XRD.

Irregular Nb_3_Sn crystals of <10 μm dimension were formed together with Nb and NbO in samples from NS-1000/60 (Figure 2a; Run 1). The presence of large Nb grains (>30 μm) indicated that an extended sintering process did not eliminate minor phases such as Nb or NbO. In Figure 2a, black areas (indicated by white arrows) indicate the presence of pores in these sintered samples. The microstructure of the NS-1000/60/arc (Figure 2b; Run 2) showed the formation of Nb and Sn phases along with Nb_3_Sn phase as well as a more consolidated microstructure with fewer pores than in Figure 2a. Most of the solidification morphologies due to arc-melting conditions possessed an approximately hemispherical shape.

Figure 2 indicates that arc melting provides higher-density products. Figure 2c clearly indicates that the Nb_3_Sn grains produced after annealing of the arc melted sample at 900 °C for 3 h (NS-720/105/arc/ann/900/3; Run 6) were significantly smaller (i.e., sub-micrometre to micrometre size) than those (i.e., >10 μm) produced at a higher temperature (1000 °C) and annealed for a longer time (60 h), as shown for NS-1000/60/arc/ann/1000/60 (Run 3). Extended annealing of the sample at the higher temperature of 1000 °C for 60 h resulted in densely packed and large grains (>50 μm) of Nb_3_Sn. This morphology was due to the significant grain growth, driven by increased atomic mobility at the higher annealing temperature over time.

**Figure 3:**
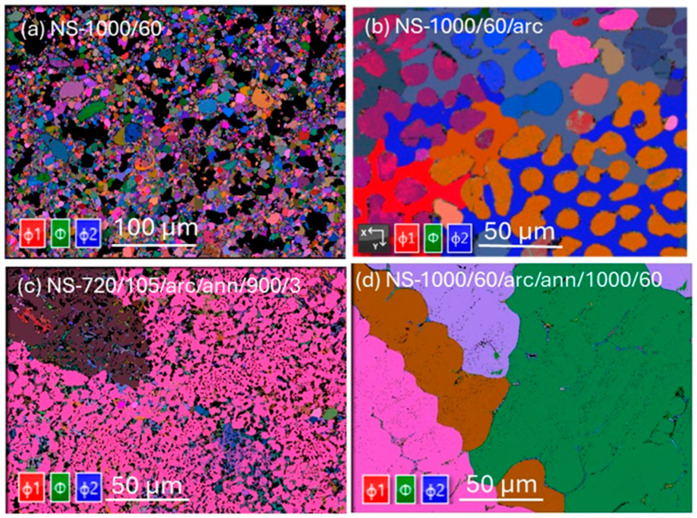
Euler maps of (**a**) NS-1000/60 (Run 1), (**b**) NS-1000/60/arc, (Run 2), (**c**) NS-720/105/arc/ann/900/3 (Run 6), (**d**) NS-1000/60/arc/ann/1000/60 (Run 3). Euler angles are characterized by rotation of φ_1_ about the z-axis (red), rotation of φ about the rotated x-axis (green), and rotation φ_2_ about the y-axis (blue). The same color signifies similar grain orientation; non-primary colors indicate intermediate orientations.

Figure 3 shows Euler maps (determined by the EBSD technique) of Nb_3_Sn samples listed in Table 2. The Euler maps, which represent the crystallographic orientation of individual grains, reveal significant variations among the Nb_3_Sn bulk samples subjected to different heat treatments. For the sintered sample NS-1000/60 (Figure 3a), the map shows a wide range of orientations, indicating a lack of common crystallographic orientation among the grains. This Euler map suggests that the sintering process at 1000 °C for 60 h did not result in a uniform texture for all grains, including Nb_3_Sn.

The sample NS-720/105/arc/ann/900/3 (Run 6) displayed a more uniform crystallographic orientation with three predominant orientations observed for all grains in the field of view (Figure 3c). This texture indicated a more consistent grain orientation resulting from the annealing process. In contrast, the sample with extended annealing at 1000 °C for 60 h after arc-melting (NS-1000/60/arc/ann/1000/60) showed the presence of large Nb_3_Sn grains, each exhibiting distinct crystallographic orientations. This grain morphology confirms that prolonged annealing at high temperatures promotes grain growth, resulting in larger grains with diverse orientations. These observations highlight the significant impact of heat-treatment protocols on the crystallographic orientation and grain structure of Nb_3_Sn samples.

**Figure 4:**
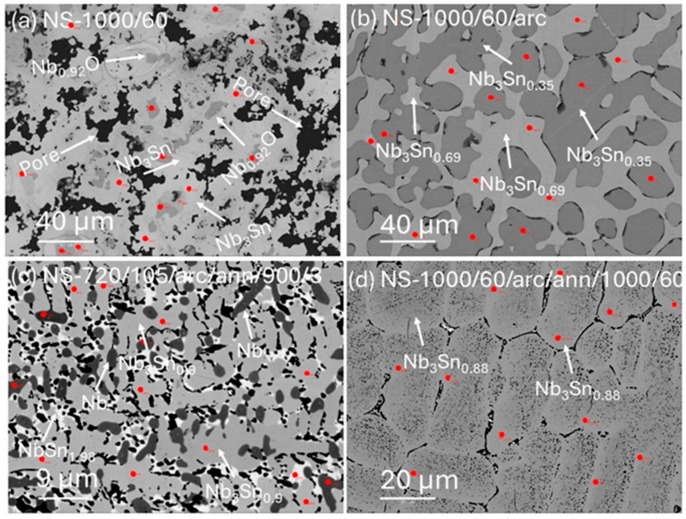
Backscattered electron images of (**a**) NS-1000/60 (Run 1), (**b**) NS-1000/60/arc (Run2), (**c**) NS-720/105/arc/ann/900/3 (Run 6) and (**d**) NS-1000/60/arc/ann/1000/60 (Run 3). The white arrows denote holes, Nb-rich, and Sn-rich areas. Red circles indicate locations of spot analyses using EPMA. Note the difference in scale bars within Figure 4.

Figure 4 shows backscattered electron (BSE) images of four samples listed in Table 1. These BSE images display a broad range of image contrast typically attributed to fluctuations in the relative mean atomic number. Generally, regions appearing lighter in a BSE image have a higher atomic number than darker regions. This image contrast highlights microstructural changes in the Nb_3_Sn samples due to different processing and annealing conditions. For instance, the black colour delineates pores in the sample (Figure 4a), while the dark grey and brighter grey colours denote the Nb-rich and Sn-rich phases, respectively. In Figure 4a, the BSE image of NS-1000/60 (Run 2) reveals Nb_3_Sn aggregate surrounded by pores. These aggregates exhibit a distinctive pattern with a dark core and a lighter outer margin, corresponding to Nb-rich and Sn-rich phases, respectively.

A BSE image of the arc-melted sample (NS-1000/60/arc; Figure 4b) shows a darker Nb-rich area surrounded by lighter Sn-rich areas (arrowed). Figure 4c shows a BSE image of NS-720/105/arc/ann/900/3 (Run 6) with uniform structure and substantially smaller grain sizes compared with NS-1000/60 (Run 1) and NS-1000/60/arc (Run 2). The BSE image in Figure 4d shows that extended annealing of the arc-melted sample at 1000 °C for 60 h (NS-1000/60/arc/ann/1000/60; Run3) resulted in larger grains (i.e., >20 μm) compared to NS-720/105/arc/ann/900/3 (Figure 4c; Run 6). The BSE images in Figure 4 were used to guide locations for EPMA analyses, the locations of which are annotated by small red dots.

Table 3 provides average compositions from selected spot analyses for syntheses identified in Table 1. For each sample, the number of point analyses (n) used to obtain average compositions based on similar BSE image contrast is also listed in Table 3, along with corresponding average analytical totals that range from 98.7 to 99.8 (wt%) for the Nb–Sn phases. A majority of individual point analyses are Nb_1−β_Sn_β_ phases, although other analyses are consistent with the presence of Nb and NbO in these samples.

In general, these data in Table 3 show that the concentration of Nb ranged from 34 to 100 at% and Sn from 10 to 66 at% across all analyses. As shown in Table 3, the stoichiometries of Nb–Sn phases included Nb_3_Sn, Nb_3_Sn_0.90_, Nb_3_Sn_0.88_, Nb_3_Sn_0.69_, Nb_3_Sn_0.35_ and NbSn_1.98_.

### 3.3. Electrical and Magnetic Properties

**Figure 5:**
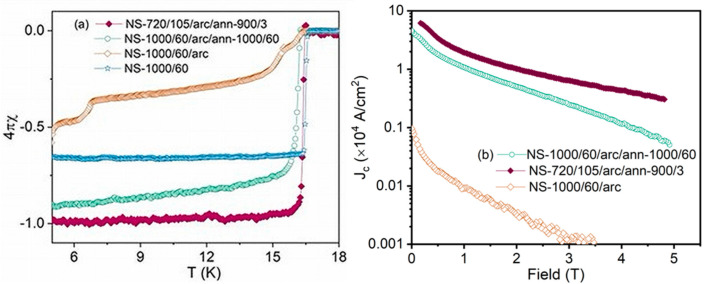
(**a**) ZFC magnetization of Nb_3_Sn from NS-1000 (blue), NS-1000/60/arc (orange), NS-720/105/arc/ann/900/3 (maroon), NS-1000/60/arc/ann/1000/60 (green) and (**b**) critical current density of the same Nb_3_Sn samples as a function of an applied magnetic field at 10 K.

Figure 5a shows the zero-field-cooled and field-cooled measurements for Nb_3_Sn bulks under an applied magnetic field of 10 Oe. All samples showed a clear superconducting transition similar to that obtained previously [14]. NS-1000/60 (Run 1) and NS-720/105/arc/ann/900/3 (Run 6) demonstrated very sharp superconducting transitions at 16.45 K and 16.58 K, respectively. NS-1000/60/arc/ann/1000/60 (Run 3) showed a slightly lower transition temperature at 16.26 K. The arc-melted sample without further annealing (NS-1000/60/arc), showed a broad transition at 16.21 K followed by other transitions at 15.42 K and 6.78 K.

Figure 5b shows the field dependence of *J*_c_ for arc-melted samples (NS-1000/60/arc; Run 2) annealed at 900 °C for 3 h (NS-720/105/arc/ann/900/3; Run 6) and annealed at 1000 °C for 60 h (NS-1000/60/arc/ann/1000/60; Run 3). As shown in Figure 5b, the *J*_c_ of arc-melted Nb_3_Sn was much lower than the *J*_c_ of post-annealed Nb_3_Sn bulks. Both Nb_3_Sn bulk samples annealed at 900 °C and 1000 °C (NS-720/105/arc/ann/900/3 and NS-1000/60/arc/ann/1000/60) demonstrated enhanced *J*_c_ performance compared with only arc-melted Nb_3_Sn (NS-1000/60/arc).

Both annealed samples, NS-720/105/arc/ann/900/3 (Run 3) and NS-1000/60/arc/ann/1000/60 (Run 6), exhibited significantly enhanced critical current density (*J*_c_), about 1.5 orders of magnitude greater compared with the arc-melted sample (NS-1000/60/arc; Run 2). NS-720/105/arc/ann/900/3 demonstrated the highest *J*_c_ across the entire field range up to 5 T, with *J_c_* = 6.16 A/cm^2^ at 0.17 T and 10 K. Specifically, at 10 K, the low-field and high-field *J*_c_ values for NS-720/105/arc/ann/900/3 (Run 6) were 1.8 times and 5.8 times higher, respectively, than comparable values for NS-1000/60/arc/ann/1000/60 (Run 3).

The range of *J_c_* values between 6.16 and 3.4 × 10^4^ A/cm^2^ for the two post-arc melt annealed samples (Run 6 and Run 3, respectively at low field) was comparable to values obtained in early experiments on Nb_3_Sn wires (e.g., produced by the bronze or PIT techniques) [7,23]. However, the *J_c_* values reported in this present study for binary bulk Nb_3_Sn were an order of magnitude less than existing wires. There are limited available data on the *J_c_* of bulk binary Nb_3_Sn obtained at 10 K.

**Figure 6:**
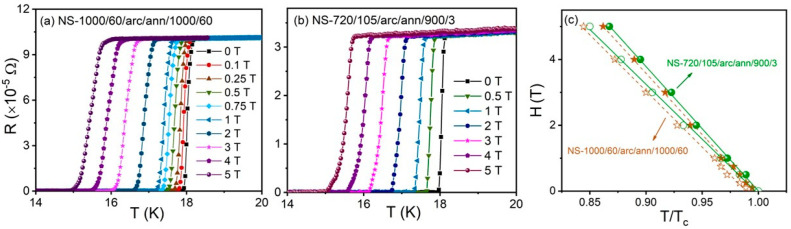
Temperature dependence of resistance for (**a**) NS-720/105/arc/ann/900/3 (Run 6) and (**b**) NS-1000/60/arc/ann/1000/60 (Run 3) under different magnetic fields up to 5 T. (**c**) H_c2_ (closed symbol) and H_irr_ (open symbol) versus temperature for NS-720/105/arc/ann/900/3 (green) and NS-1000/60/arc/ann/1000/60 (orange).

Electrical resistance, R, versus temperature, T, curves for samples from NS-720/105/arc/ann/900/3 (Run 6) and NS-1000/60/arc/ann/1000/60 (Run 3) in the presence of magnetic fields up to 5 T are shown in Figure 6a,b. Electrical resistance started to drop toward zero at T_c_= 18.0 K in zero magnetic field for both samples. This value aligns well with reports for the onset of superconductivity at 18.05 K ± 0.1 reported by Matthias et al. [1]. The onset of T_c_ decreased with the increasing magnetic field, while the transition width, ΔT_c_, increased from 0.1 K to 0.4 K for both samples, as shown in Figure 6a,b.

Figure 6c shows estimates for the upper critical field, *H*_c2_, defined by *R*(*T*, *H*c_2_) = 0.9*R*_n_, representing the normal state resistance measured just above the onset of the critical temperature. Similarly, the irreversibility field, *H*_irr_, is defined by R(*T*,*H*_irr_) = 0.1*R*_n_ and is also shown in Figure 6c. From Figure 6c, we calculated slopes of 2.09 and 1.89 for dH_c2_/dT and dH_irr_/dT, respectively, for NS-720/105/arc/ann/900/3 (Run 6). Similarly, NS-1000/60/arc/ann/1000/60 (Run 3) yielded values of 2.05 and 1.85 T/K for dH_c2_/dT and dH_irr_/dT.

The *H*_c2_ (0) was estimated using the Werthamer–Helfand–Hohenberg formula, *H*_c2_ = −0.69*T*_c_(d*H*_c2_/d*T*), with d*H*_c2_/d*T* at *T* = *T*_c_. Using this formula, values for *H*_c2_(0) = 26.1 and 25.6 T were obtained for the sample from NS-720/105/arc/ann/900/3 (Run 6) and NS-1000/60/arc/ann/1000/60 (Run 3), respectively. These values were lower than the reported value of 28.7 T for 23.6 ± 0.7 at.% Sn composition variation, noting that bulk samples in the study by Jewell et al. [24] were obtained using HIP techniques at 1020 °C on powder-in-tube samples, followed by post-HIP heat treatment at 1800 °C for 24 h.

## 4. Discussion

In this work, we describe combinations of sintering, arc-melting, and annealing procedures that affect the morphology, stoichiometry and superconducting properties of bulk Nb_3_Sn samples. Sintering of Sn and Nb is undertaken prior to arc melting in order to mitigate Sn evaporation at higher temperatures. The Nb–Sn phase diagram indicates that the superconducting Nb_1−β_Sn_β_ phase occurs within the range for 0.18 < β < 0.25 for which 6 K < T_c_ < 18 K [5]. This phase can be synthesized either above 930 °C in the presence of a Sn–Nb melt or below this temperature through solid-state reactions involving Nb and Nb_6_Sn_5_ or NbSn_2_ [12].

### 4.1. Structure and Composition

The formation of Nb_3_Sn was not favourable for the sample sintered at 720 °C for 105 h because the diffusion of Nb and Sn was relatively slow. However, Nb_3_Sn was a primary phase of the sample sintered at 1000 °C (NS-1000/60) prior to arc melting, according to XRD and EBSD phase analysis. A higher sintering temperature of 1000 °C prior to arc melting promoted faster diffusion and effective transformation of the starting mixture. After arc melting, the sample showed a high proportion of Nb_3_Sn phase and the presence of residual Nb and Sn (as shown in Figure 2b, Figure 3b and Figure 4b), albeit with a lower proportion of Nb_3_Sn compared with Run 1 or Run 3. In addition, EPMA analyses (Table 3) show that individual grains are marginally within the superconducting range for Nb_1−β_Sn_β_ phases (i.e., β =18.6 (1)).

Post-annealing serves as an important step to stabilize metastable phases that form during quenching and facilitates the formation of the target composition. In both cases, for Run 3 and Run 6, annealing after arc melting resulted in compositions of Nb_1−β_Sn_β_ phases within the superconducting range [5]. However, a substantial difference in grain size was observed between the samples. Annealing of NS-720/105/arc/ann/900/3 (Run 6) at 900 °C yielded a uniform microstructure with smaller (<5 μm) grain sizes.

A uniform microstructure (i.e., similar grain orientation) influences macroscopic mechanical properties such as elasticity and strain distribution as well as critical current [25]. This influence competes with an increased density of grain boundaries in bulk polycrystalline samples of Nb_3_Sn, such as from Run 6. Smaller grain size, to the sub-micrometer scale, also results in substantial improvement to *J_c_* values in wires produced in ternary systems such as Nb_1−β_Sn_β_X (where X = Cu, Ti, Ta, Zr) [23,26,27]. We suggest that the same principles apply to production of bulk binary Nb_3_Sn.

**Figure 7:**
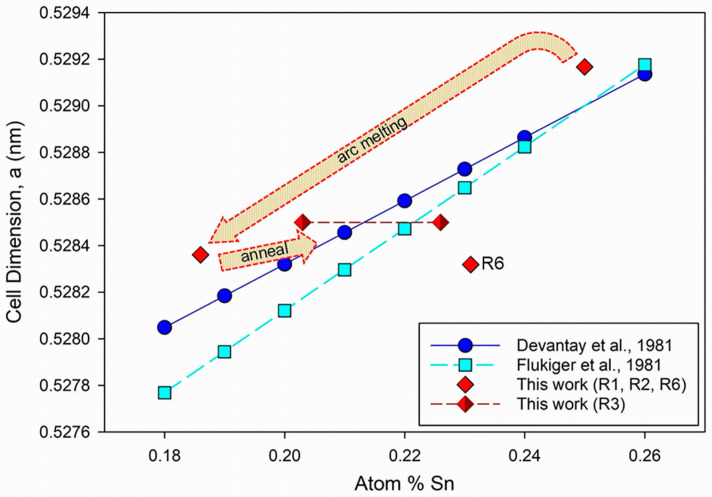
Refined cell dimensions plotted against Sn content for Nb_1−β_Sn_β_ samples listed in Table 3 (red diamonds). Linear plots for Nb_1−β_Sn_β_ obtained in other studies ([8] blue solid line; [9] cyan dotted line); The trajectory by [8] is preferred by Goedeke [5]. Estimated standard errors for data from this study are within the symbol size. Note the statistically equivalent number of compositions for samples from Run 3, which straddle the trajectories determined by Devantnay et al. [8] and Flükiger et al. [9]. Adapted from Figure 3 of Goedeke [5] with permission.

Goedeke [5] provided a comprehensive and informative review of early experiments on production of homogenous Nb_3_Sn, noting that the general variation of superconducting properties for A15 compounds consistently depended on cell dimension, atomic content (for Nb_1-β_Sn_β_, the value of β), normal state resistivity, and long-range order. Figure 7 is an adaptation of Figure 3 in [5], showing the variation of lattice parameter, *a*, with atomic content of Sn in Nb_1−β_Sn_β_. Figure 7 includes average data for individual grains within the four samples that showed compositions within the range 0.18 < β < 0.25 (Table 3).

Figure 7 suggests that pre-treatment of Nb–Sn ingots before arc melting combined with post-treatment annealing may enable production of bulk Nb_1−β_Sn_β_ with a targeted stoichiometry. For example, Rietveld (Table 2) and EPMA compositional (Table 3) analyses show that Run 1 produced a predominantly β = 0.25 compound with pre-treatment. Arc melting of this sample produced a mixture with lower proportions of Nb_1−β_Sn_β_ with β = 0.186 and substantial Nb. Annealing after arc melting resulted in a sample with high proportions of two Nb_1−β_Sn_β_ compounds (Table 3) with β = 0.226 and β = 0.203. These trends are shown in Figure 7 by the broad dotted red arrows and suggest that further attention to annealing times post-arc melting could result in a bulk single-phase Nb_1−β_Sn_β_ compound.

The optimum annealing times and appropriate temperatures to maximize single-phase Nb_3_Sn remains uncertain based on data obtained to date. Nevertheless, the example of Run 6, from which predominantly Nb_1−β_Sn_β_ formed with β = 0.231 after a lower pre-treatment temperature and very short annealing time, implies that within the extremes of processing represented by Run 3 and Run 6, evaluation of these parameters indicates potential for scalable production of bulk Nb_3_Sn. In the latter case, smaller micrometer-scale grain size and enhanced *J*_c_ compared with other samples in this study suggest that annealing times of 3 h < t_post_ < 60 h at temperatures between 900 °C and 1000 °C are viable targets for future experiments. Future processing and microstructural studies would benefit from experimental determination of macroscopic properties [25] and electronic property calculations informed by defect identifications [28], as well as in situ XPS techniques [29], scanning Hall probe microscopy [30] and a range of Synchrotron experiments [31,32,33].

### 4.2. Superconductivity

The arc-melted sample for NS-1000/60/arc (Run 2) exhibited a wide superconducting transition at 16.21 K, accompanied by additional transitions at 15.42 K and 6.78 K. The low *T*_c_ = 6.78 K could have been due to formation of a solid solution with lower *T*_c_ associated with lower Sn content (β = 0.104) as indicated by EPMA data (see Table 3). It has been reported that at low concentrations (β < 0.05 for Nb_1-β_Sn_β_), the incorporation of Sn into Nb forms a solid solution that lowers the *T*_c_ of Nb from 9.2 K to approximately 4 K for β = 0.05 [5,12,34].

In the A15 Nb_3_Sn structure, which had a lattice parameter of about 0.529 nm for the stoichiometric composition [8], the separation between Nb atoms was roughly 0.265 nm. Reduced distance between Nb atoms in the chains is believed to create a confined peak in the d-band density of states (DOS), resulting in a notably increased DOS near the Fermi level. This enhancement is thought to play a role in the higher critical temperature (*T*_c_) of Nb_3_Sn compared to other Nb-based superconductors. Additionally, it has been observed that excess Nb atoms can occupy Sn sites, leading to anti-site disorder [35].

Ab initio density functional theory (DFT) calculations by Besson et al. [22] reveal that Nb_3_Sn essentially appears as an anti-site compound, with the density of vacancies, particularly Sn vacancies, remaining negligible below the melting temperature [36]. Additionally, an EXAFS study examining the occupancy of Ta/Ti sites in Nb_3_Sn wires revealed that the top-performing Ti-doped strand was considerably sub-stoichiometric in Sn [37]. This anti-site disorder is likely to be responsible for the high average behaviour of the upper critical field (Hc2). EPMA analyses (Table 3) showed non-stoichiometric compositions in samples from Runs 2, 3, and 6; implicit confirmation that the density of vacancies and/or anti-site disorder influence electronic properties of Nb_1−β_Sn_β_ compounds, as noted previously [5]. These findings as well as detailed computational studies [38] suggest that dopants and anti-site disorder play a crucial role in minimizing the variability of superconducting properties and maximizing high-field *J*_c_ properties.

Table 4 shows a comparison between experimental data and calculated *T*_c_ using DFT from the work of Carlson et al. [39] and *T*_c_ values obtained for samples in this work. The Sn concentration according to EPMA results (Table 3) can be correlated with the experimental determination of T_c_ [20]. For both the DFT calculations and the experimental results in our work, *T*_c_ values increased with increasing Sn concentration. The calculated T_c_ and the Fermi-level DOS showed strong agreement across all Sn concentrations [39]. The presence of Sn anti-site defects is believed to significantly broaden a reduction in the density of states (DOS) at the Fermi level near grain boundaries [39]. This presence of anti-site defects may explain the reduction in *T*_c_ from 16.58 K for Nb_3.00_Sn (NS-1000/60; Run 1) to 16.25 K for Nb_3_Sn_0.88_ + Nb_3_Sn_0.77_ (NS-1000/60/arc/ann/1000/60; Run 3).

The *J*_c_ of a bulk superconductor in a magnetic field is governed by the pinning force (F_p_) generated by appropriately sized crystal defects that hinder the movement of magnetic vortices. The efficiency of pinning is highest when defects approach the coherence length of the superconductor, typically 2–4 nm for Nb_3_Sn [40]. Defects smaller or larger than this size result in reduced pinning efficiency. Grain boundaries, which are the regions where different crystal grains meet, can also act as pinning centres for magnetic flux lines. The significant impact of grain boundaries on the superconducting properties of Nb_3_Sn is widely recognized [41,42]. These boundaries play a crucial role in pinning vortices and determining critical currents in Nb_3_Sn wires, as documented in previous studies [5,43,44] and experimentally demonstrated using atom probe tomography combined with *J*_c_ measurements on Nb_3_Sn wires synthesized by the internal tin process [41].

The enhanced *J*_c_ of the bulk sample from NS-720/105/arc/ann/900/3 (Run 6) related to the formation of smaller grains (i.e., <5 μm) and the higher density of grain boundaries compared with the arc melted and post-annealed Nb_1−β_Sn_β_ sample synthesized at higher temperature. The NS-1000/60 (Run 1) and NS-1000/60/arc (Run 2) with large grains showed a lower *J*_c_ compared with NS-720/105/arc/ann/900/3 (Run 6), especially at higher magnetic fields.

Simulation by Carlson et al. [39] demonstrated that grain boundaries serve a dual role, acting as nucleation points for vortex entry and as pinning sites once vortices have formed. The behaviour of the vortices depends on the strength of the applied field—vortices can either stay pinned at the grain boundaries or pass into the grains. This interpretation is consistent with higher supercurrent loss for NS-1000/60/arc/ann/1000/60 (Run 3) compared with NS-720/105/arc/ann/900/3 (Run 6) for H > 2 T. Additionally, observations have revealed compositional variations within Nb_3_Sn grain boundaries at a nanometer scale [45,46] that affect the supercurrent performance of Nb_3_Sn superconducting wires. Among the samples studied, NS-720/105/arc/ann/900/3 (Run 6) with an average Nb_3_Sn_0.9_ composition showed the highest supercurrent performance. This performance, as noted above, was due to the high density of grain boundaries (Figure 3c and Figure 4c) and may have been additionally enhanced by the presence of anti-site defects.

## 5. Conclusions

This investigation explored the impact of synthesis procedures using pre-treatment, arc melting, and post-arc-melt annealing on the morphology, grain boundary formation, and stoichiometry of binary Nb_1−β_Sn_β_ bulk superconductors. Arc-melted the sample annealed at 900 °C for 3 h (β = 0.231) resulted in enhancement of *T*_c_ and *J*_c_ compared with other samples in this work. This enhancement was attributed to recrystallization of smaller grains and formation of effective pinning centres such as a higher proportion of grain boundaries and anti-site defects. However, annealing at 1000 °C for 60 h after arc melting reduced *J*_c_. A decrease in *T*_c_ from 16.58 K for Nb_3.00_Sn in a sintered binary sample to 16.25 K for an arc-melted and annealed sample was attributed to the production of two Nb_1−β_Sn_β_ compounds (β = 0.226 and β = 0.203) with a large grain size (>50 μm) and no Nb. The success of these two alternate synthesis procedures involving pre-treatment sintering, followed by arc melting and subsequent annealing at high temperature (>900 °C) for different times suggest that further exploration of sintering and annealing conditions offers potential for scalable production of bulk Nb_1−β_Sn_β_ compounds of predictable stoichiometry.

## Figures and Tables

**Table 1:** Sample ID, synthesis technique and heat treatment conditions for Nb_3_Sn samples.

Run	Sample ID	Sintering Condition	Arc-Melt	Post Arc-Melt Heat Treatment
1	NS-1000/60	1000 °C/60 h	No	No
2	NS-1000/60/arc	1000 °C/60 h	Yes	No
3	NS-1000/60/arc/ann/1000/60	1000 °C/60 h	Yes	1000 °C/60h
4	NS-720/105	720 °C/105 h	No	No
5	NS-720/105/arc	720 °C/105 h	Yes	No
6	NS-720/105/arc/ann/900/3	720 °C/105 h	Yes	900 °C/3h

**Table 2:** Relative phase proportions and lattice parameters determined by Rietveld refinements.

Run	Sample ID	Nb_3_Sn (%)	NbO (%)	Nb (%)	Unit Cell * (Å)	R_wp_ (%)	gof
1	NS-1000/60	94.69	3.57	1.74	5.29167(3)	13.8	1.5
2	NS-1000/60/arc	85.16	4.80	10.04	5.28360(5)	13.5	1.6
3	NS-1000/60/arc/ann/1000/60	94.88	5.12	-	5.28510(4)	12.2	1.5
6	NS-720/105/arc/ann/900/3	85.99	3.49	10.52	5.28318(6)	12.5	1.2

* Estimated standard deviation in parentheses to the least significant figure.

**Table 3:** Average compositions of selected grains in Nb–Sn samples determined by EPMA.

Sample	n **	Element Atom %				Totals (wt%)	Stoichiometry		
		Nb	Sn	O	Total		Nb	Sn	O
NS-1000/60 (R1)									
Nb_3_Sn_1.00_	21	75.0 (2)	25.0 (2)	-	100	98.7 (4)	3	1.00 (1)	-
Nb_0.92_O	9	47.9 (7)	-	52.1 (7) *	100	100	0.92	-	1
Nb	5	100.0	-	-	100		1	-	-
NS-1000/60/arc (R2)									
Nb_3_Sn_0.69_	20	81.4 (1)	18.6 (1)	-	100	99.6 (3)	3	0.69 (1)	-
Nb_3_Sn_0.35_	20	89.6 (2)	10.4 (2)	-	100	99.5 (2)	3	0.35 (1)	-
NS-1000/60/arc/ann/1000/60 (R3)									
Nb_3_Sn_0.88_	9	77.4 (6)	22.6 (6)	-	100	99.8 (2)	3	0.88 (3)	-
Nb_3_Sn_0.77_	10	79.7 (4)	20.3 (4)	-	100	99.7 (3)	3	0.77 (2)	-
NS-720/105/arc/ann/900/3 (R6)									
Nb_3_Sn_0.90_	21	76.9 (3)	23.1 (3)	-	100	99.8 (2)	3	0.90 (2)	-
Nb_1_Sn_1.98_	5	33.6 (4)	66.4 (4)	-	100	98.7 (4)	1	1.98 (3)	-

* Oxygen calculated by difference from 100; ** n is the number of point analyses used to obtain average composition; values in parentheses are one standard deviation to the least significant figure. Weight percentage totals provide an indication of analytical precision.

**Table 4:** Calculated *T*_c_ versus measured T_c_ for different concentrations of Sn in Nb_1-β_Sn_β_.

Sn (at %) (Calc) [39]	*T*_c_ (K) (Calc) [39]	*T*_c_ (K) (Experiment) [39]	Sn (at %) (This Work)	*T*_c_ (K) (This Work)
18.75	9.2	6	18.60	6.78 *
20.83	11.3	9.5	22.59	16.26 *
23.44	16.1	16	23.09	16.45 *
25.00	18.2	18	25.02	16.58 *

* *T*_c_ obtained from ZFC and FC measurements.

## Data Availability

The original contributions presented in this study are included in the article. Further inquiries can be directed to the corresponding author. Comprehensive EPMA results can be provided upon request.
